# Cathodal Occipital tDCS Is Unable to Modulate the Sound Induced Flash Illusion in Migraine

**DOI:** 10.3389/fnhum.2019.00247

**Published:** 2019-07-17

**Authors:** Simona Maccora, Giuseppe Giglia, Nadia Bolognini, Giuseppe Cosentino, Massimo Gangitano, Giuseppe Salemi, Filippo Brighina

**Affiliations:** ^1^Department of Biomedicine, Neuroscience and Advanced Diagnostics (BIND), University of Palermo, Palermo, Italy; ^2^Department of Psychology & NeuroMi Milan Center for Neuroscience, University of Milan Bicocca, Milan, Italy; ^3^Laboratory of Neuropsychology, IRCSS Istituto Auxologico Italiano, Milan, Italy; ^4^Department of Brain and Behavioral Sciences, University of Pavia, Pavia, Italy; ^5^Clinical Neurophysiology Unit, IRCCS Mondino Foundation, Pavia, Italy

**Keywords:** migraine, tDCS, Sound-induced Flash Illusion, visual cortex, pain

## Abstract

Migraine is a highly disabling disease characterized by recurrent pain. Despite an intensive effort, mechanisms of migraine pathophysiology still represent an unsolved issue. Evidence from both animal and human studies suggests that migraine is characterized by hyperresponsivity or hyperexcitability of sensory cortices, especially the visual cortex. This phenomenon, in turn, may affect multisensory processing. Indeed, migraineurs present with an abnormal, reduced, perception of the Sound-induced Flash Illusion (SiFI), a crossmodal illusion that relies on optimal integration of visual and auditory stimuli by the occipital visual cortex. Decreasing visual cortical excitability with transcranial direct current stimulation (tDCS) can increase the SiFI in healthy subjects. Moving away from these issues, we applied cathodal tDCS over the visual cortex of migraineurs, with and without aura, in order to decrease cortical excitability and thus physiologically restoring the perception of a reliable SiFI. Differently from our expectations, tDCS was unable to reliably modulate SiFI in migraine. The chronic, relatively excessive, visual cortex hyperexcitability, featuring the migraineur brain, may render tDCS ineffective for restoring multisensory processing in this disease.

## Introduction

Migraine is a disease characterized by recurrent headache attacks with throbbing, moderate to severe pain, associated with nausea and/or vomiting, and intolerance to light and sounds. Migraine hits part of the general population and severely impacts quality of life; it is indeed the third cause of disability in the general population (Stovner et al., 2007; Saylor and Steiner, 2018).

Mechanisms underlying migraine attacks, including activation and sensitization of the trigeminovascular pathway (Bernstein and Burstein, 2012) and the involvement of the pain matrix (Schulte and May, 2016), remain largely unknown (Dodick, 2018).

Non-Invasive Brain Stimulation (NIBS) studies have revealed that cortical hyperexcitability is a key marker of the pathophysiology of migraine, influencing the state of striate (Aurora et al., 1999; Palermo et al., 2009; Brighina et al., 2011) extrastriate cortices (Fierro et al., 2003), and of the motor cortex (Áfra et al., 1998; Brighina et al., 2011). Changes in cortical excitability seem to occur according to the migraine phase (Cosentino et al., 2014). Neuroimaging evidence also supports the hypothesis of visual cortex hyperexcitability (de Tommaso et al., 2017).

Recently, a behavioral paradigm, the Sound-induced Flash Illusion (SiFI; Shams et al., 2002; de Tommaso et al., 2017), has been used to investigate visual cortical excitability and multisensory processing in healthy humans and migraineurs (Bolognini et al., 2011; Brighina et al., 2015). The SiFI is a crossmodal perceptual illusion by means of which a single flash accompanied by multiple beeps gives rise to the illusory perception of seeing multiple flashes, according to the beeps number (fission phenomenon). Such illusory effect emerges from visual-auditory interactions within the occipital cortex (Shams et al., 2005). Brighina et al. (2015) found that migraineurs report less fission effects than healthy subjects, suggesting that the visual cortical hyperexcitability of migraine may affect multisensory interactions driving the SiFI. In healthy individuals, the fission illusion is decreased by increasing occipital excitability with anodal transcranial direct current stimulation (tDCS), while it is increased by occipital cathodal tDCS (Bolognini et al., 2011). The fusion phenomenon, where a single beep paired with multiple flashes reduces the number of seeing flashes, is unaltered in migraine, and not reliably modulated by tDCS in healthy individuals (Bolognini et al., 2011; Brighina et al., 2015).

On such basis, we hypothesize that following the reverse path, namely reducing visual cortical excitability, should normalize SiFI perception in migraine. Moving from this hypothesis, cathodal (putatively inhibitory) tDCS was applied over the visual cortex of migraine patients in order to restore their physiologically susceptibility to the fission phenomenon.

## Materials and Methods

### Participants

Twenty-two right-handed patients with episodic migraine (11 with aura, MwA, mean age 28.9 SD 10.5; five male and six females 11 without aura, MwoA; mean age 33.2 ± SD 10.5; four male and seven females) were consecutively enrolled at the outpatient Headache Center at the University of Palermo. Mean age was not significantly different between groups (*p* = 0.35) Mean number of attacks/month were 3.1 (±1.4) in MwoA and 2.9 (±1.3) in MwA. Diagnosis was made by expert neurologists according to the International Classification of Headache Disorders [ICHD-3; Headache Classification Committee of the International Headache Society (IHS), (2018)]. All patients were not taking any prophylaxis drugs and no other than symptomatic medications were allowed during the week before experiments, but they have the possibility to use their analgesic symptomatic drug in the case of a migraine attack. The experimental procedure was carried out during the interictal period (i.e., at least 48 h since last migraine attack). The study was approved by the Ethical Committee “Palermo 1”of the Policlinico Paolo Giaccone, University of Palermo. All patients gave their informed consent to the study.

### Stimuli

The same experimental procedure by Brighina et al. (2015) was used. Participants were seated in a noise-protected and slightly dimmed room at about 57 cm from a standard CRT monitor (Samsung SyncMaster 1200NF: resolution 1,024 × 768, refresh rate 75 Hz) with their head supported by a chin-rest. The subject’s seat was positioned so that eye level was at the middle of the display monitor that was centered on his/her sagittal midplane. Visual stimuli consisted of a white disk (luminance = 118 cd/m^2^) subtending 2° at the eccentricity of 5° of visual field. Auditory stimuli were beeps (80 dB sound pressure level) coming from a pair of speakers positioned near the screen and aligned with the flashes. Each trial was preceded by the appearance of a white fixation cross, displayed at the center of the black screen (luminance = 0.02 candela cd/m^2^). Stimuli were presented electronically using the E-Prime 2.0 software (Psychology Software Tools, Pittsburgh, PA, USA).

**Figure 1:**
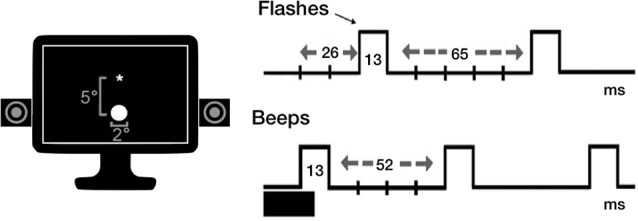
Overview of the stimuli used for SiFI task.

The task comprised 11 experimental conditions: single flash trials (1F), accompanied by 0–4 beeps (B; i.e., 1F0B, 1F1B, 1F2B, 1F3B, 1F4B) to induce the fission illusion; multiple flash trials (i.e., 2F, 3F, 4F), accompanied by 0 or 1 beep (2F0B, 3F0B, 4F0B, 2F1B, 3F1B, 4F1B) for the fusion illusion. Each flash and beep lasted one screen refresh (13 ms). The first flash appeared 26 ms after the first beep. The stimulus onset asynchrony was of 65 ms (five refreshes) between flashes, and of 52 ms (four refreshes) between beeps (see Figure 1). Eight trials were presented in random order for each experimental condition (total number of trials = 88; task duration of approximately 5 min). In each trial, we instructed participants to report the number of seen flashes. Ten practice trials (not considered in the analyses) were presented as a short familiarization procedure before the beginning of the task.

### tDCS

tDCS was applied *via* a pair of saline-soaked surface sponge electrodes (35 cm^2^) and delivered by a CE-certified Eldith DC stimulator (Neuroconn, Ilmenau, Germany). A monopolar montage was used, with the cathode over the occipital cortex (Oz of the 10–20 EEG system) and the anode (reference electrode) over Cz (Antal et al., 2011). The current was ramped up during the first 10 s to a maximum of 2 mA, and then remained for the 10-min stimulation period, that was initiated 5 min before stimuli presentation. The current density was of 0.06 mA/cm^2^ over the stimulated area. In the sham condition, the stimulator was turned off after 30 s causing the same itching sensation as experienced with the real stimulation, but without having any biological effects (Giglia et al., 2011). The choice of stimulation duration was based on literature data showing that 9 min of tDCS induces after-effects lasting up to 30 min (Nitsche and Paulus, 2001). All subjects underwent, in a random order, three experimental sessions (at least 7 days apart): baseline (i.e., without tDCS), cathodal and sham tDCS.

### Statistical Analysis

According to a previous study reporting significant effect of tDCS (Bolognini et al., 2011), we used a similar sample size.

Continuous variable data were collected and repeated-measures analysis of variance (rmANOVA) were performed to assess both fission and fusion illusions. For fission illusion, the mean perceived flashes in 1-flash (1F) trials were analyzed with the between-subjects factor Group (MwA vs. MwoA), and the within-subjects factors Beep (0–4 beeps) and Session (baseline, cathodal tDCS, sham tDCS). For the fusion illusion, mean perceived flashes were analyzed with the between-subjects factor Group, and the within-subjects factors Beep (0–1), Flash (2–4) and Session. Bonferroni test was used for multiple comparisons. *P*-value was considered significant at 0.05 level.

## Results

**Figure 2:**
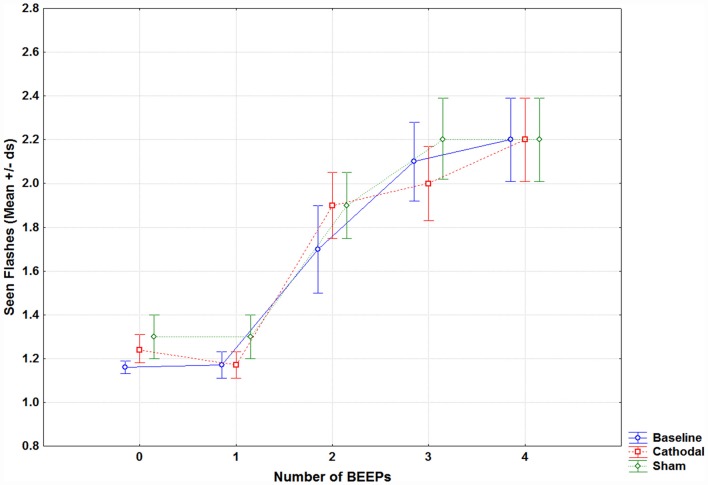
Mean seen flashes in fission trials (1 flash combined with 0 to 4 beeps) reported by patients with migraine in the baseline (without tDCS, blue line), during cathodal occipital (red line) and sham tDCS (green line).

Results were not influenced by age, as analysis of covariance (ANCOVA) did not show any significant variation for all parameters (all *p* > 0.05). Results for each condition are reported in Supplementary Table S1. Results showed a main effect of Beep (*F*_(4,80)_ = 51.43, *p* < 0.001) and a significant Session × Beep interaction (*F*_(8,160)_ = 2.23, *p* = 0.02; see Figure 2). *Post hoc* comparisons showed no differences between baseline and the sham session variables (a correction analysis in order to consider that a possible non-normal distribution of data was performed). Mauchly Sphericity Test was significant (*p* < 0.01), thus a Greenhouse-Geisser correction was run. The corrected p still remained significant (*p* = 0.03, epsilon 0.57). The only significant difference was found between 1F2B in baseline and in sham tDCS (*p* < 0.001).

For the fusion illusion, results showed the main effects of Beep (*F*_(1,20)_ = 11,071, *p* < 0.001), Flash (*F*_(2,40)_ = 692.3, *p* < 0.001), Group (*F*_(1,20)_ = 5.18, *p* = 0.03) and Session (*F*_(2,40)_ = 3.25, *p* = 0.04); for the latter, multiple comparisons showed that the baseline was different from both cathodal (*p* = 0.03) and sham (*p* = 0.03) sessions. The significant difference between groups was due to a slightly higher number of perceived flashes by MwA patients (mean = 2.89), as compared to MwoA patients (mean = 2.57, *p* = 0.03). No other significant effect was found.

Additional analyses were performed in order to compare the present findings to data of 24 healthy individuals taken from the study by Brighina et al. (2015). This exploratory analysis should be considered in light of the different age of the participants (mean age of healthy controls = 42 ± 17), which is known to affect the perception of the SiFI (e.g., McGovern et al., 2014; Bolognini et al., 2016). We analyzed only the fission effect, shown to be different in migraine (Brighina et al., 2015) and modulable by tDCS (Bolognini et al., 2011), considering migraine patients as a single, given the previous findings, which did not show different fission effects in MwA and MwoA. Separate rmANOVA were performed on fission illusion comparing migraineurs vs. controls in baseline (*F*_(4,176)_ = 2.43, *p* = 0.048; effect size: partial *η*^2^ = 0.05).

## Discussion

We chose to apply the 2 mA cathodal tDCS because the evidence of consistent intra- and inter-individual results increases with the employed current of stimulation. Modulation of M1 excitability shows a good degree of reliability across individuals and the induced effects were consistent on different days for repeated tDCS sessions (López-Alonso et al., 2015; Ammann et al., 2017). Moreover, we added a control condition (sham) that tested the possible inter-individual variability of cortical excitability.

The present results confirm a reduced fission illusion in migraineurs, in line with literature (Brighina et al., 2015). Cathodal occipital tDCS does not restore this crossmodal illusion in migraine, exerting mostly an unspecific modulation, likely due to a placebo effect (i.e., the difference between baseline and both sham and cathodal tDCS). MwA patients perceive more flashes (regardless of the number of sounds) than MwoA patients; such higher visual responsivity in MwA may be interpreted as a behavioral landmark of the phenomenon of cortical spreading depression that features the migraine visual aura, which consists in a wave of neuronal and glial depolarization, followed by long-lasting suppression of neural activity in the visual cortex (Bowyer et al., 2001). Other clinical factors, such as drugs intake and the frequency of migraine attacks, may also play a role in shaping visual reactivity of MwA patients.

Overall, this study indicates that cathodal tDCS is unable to physiologically modulate the SiFI in migraineurs. A possible explanation could be found on the general lack of efficacy of cathodal tDCS on cortical excitability in migraineurs. Indeed, although cathodal tDCS has been used as a prophylactic treatment in migraine patients, its clinical effectiveness in reducing migraine attacks was found to be independent of changes in cortical excitability (Antal et al., 2011); even if applied over consecutive sessions, cathodal tDCS cannot decrease visual cortical excitability, with 3 out of 10 patients showing even a paradoxical increase in it (Rocha et al., 2015). Similar findings have been obtained with low-frequency repetitive Transcranial Magnetic Stimulation (Brighina et al., 2002).

The lack of differences in multisensory perception following tDCS in migraine, at variance to the significant modulation reported in healthy conditions (Bolognini et al., 2011), suggests that a state of altered metaplasticity in migraine (Cosentino et al., 2014) could abolish the perceptual effects of tDCS-induced cortical excitability shift. While the slightly different stimulation parameters from a previous study (Bolognini et al., 2011) should be taken into account in order to explain the results, this seems to be unlikely, owing to the fact that we used a longer stimulation period (10 vs. 8 min), keeping other parameters unchanged. Alternatively, we may speculate that cathodal tDCS is ineffective due to the abnormal state of visual cortex hyperexcitability of migraine; this state would lead to an impaired multisensorial integration in the migraineur brain, which cannot be reversed by a single tDCS session.

Another potential explanation is that the impaired multisensorial integration is not directly caused by the visual cortex hyperexcitability itself. In healthy subjects, there is an optimal time window (about ±115 ms) for allowing audio-visual interactions giving rise to the SiFI, consistent with the multisensory integration time of superior colliculus neurons (Meredith et al., 1987). However, the migraineur brain has dysfunctional thalamocortical oscillatory networks, which impair the temporal activation of different neuronal cortical networks (de Tommaso et al., 2014). This mechanism fits well with the known deficit of habituation of the migraineur cortex (Schoenen et al., 1995; Brighina et al., 2009), which relies on integration of unimodal stimuli rhythmically presented (at fixed time intervals; Dimitrijević et al., 1972). A similar mechanism may explain the present finding: the SiFI reflects an efficient multisensory processing, which requires an optimal “phase-resetting” of ongoing oscillatory activity (van Atteveldt et al., 2014). In this view, a dysfunctional thalamocortical circuit in migraineurs could also play a main role in the abnormal SiFI perception.

## Ethics Statement

All subjects gave written informed consent in accordance with the Declaration of Helsinki. The protocol was approved by the ethical committee “Palermo 1” of the Policlinico Paolo Giaccone, University of Palermo.

## Author Contributions

SM, GG and FB contributed to the conception and design of the study. SM, GC and MG performed the experiments and collected the data. GG and NB performed the statistical analysis and interpreted the data. GG wrote the first draft of the manuscript. GS, NB and FB critically revised the manuscript. All authors contributed to the manuscript revision, read and approved the submitted version.

## Conflict of Interest Statement

The authors declare that the research was conducted in the absence of any commercial or financial relationships that could be construed as a potential conflict of interest.
