# Supplementary material for: Cathodal Occipital tDCS Is Unable to Modulate the Sound Induced Flash Illusion in Migraine
Source: Front Hum Neurosci. 2019 Jul 17;13:247. doi: 10.3389/fnhum.2019.00247 (PMC6650581; doi:10.3389/fnhum.2019.00247)
Supplement: Supplementary file 1 [file Table_1.DOC]

| **A- Fission illusion** | | | | | | | | | | | | | | | | |
| --- | --- | --- | --- | --- | --- | --- | --- | --- | --- | --- | --- | --- | --- | --- | --- | --- |
|  |  | | | | |  | | | | |  | | | | |  |
| **MwA** | **Baseline** | | | | | **Cathodal** | | | | | **Sham** | | | | | |
| Flashes/Beeps | **1/0** | **1/1** | **1/2** | **1/3** | **1/4** | **1/0** | **1/1** | **1/2** | **1/3** | **1/4** | **1/0** | **1/1** | **1/2** | **1/3** | **1/4** |  |
| Perceived flashes | 1.17 | 1.23 | 1.75 | 2.08 | 2.14 | 1.38 | 1.25 | 1.92 | 2.14 | 2.35 | 1.45 | 1.35 | 2.02 | 2.29 | 2.21 |  |
| *sd* | *0.07* | *0.06* | *0.12* | *0.18* | *0.21* | *0.09* | *0.07* | *0.15* | *0.18* | *0.21* | *0.09* | *0.07* | *0.15* | *0.20* | *0.22* |  |
|  |  |  |  |  |  |  |  |  |  |  |  |  |  |  |  |  |
| **MwoA** |  |  |  |  |  |  |  |  |  |  |  |  |  |  |  |  |
| Flashes/Beeps | **1/0** | **1/1** | **1/2** | **1/3** | **1/4** | **1/0** | **1/1** | **1/2** | **1/3** | **1/4** | **1/0** | **1/1** | **1/2** | **1/3** | **1/4** |  |
| Perceived flashes | 1.15 | 1.11 | 1.68 | 2.11 | 2.29 | 1.13 | 1.10 | 1.81 | 1.93 | 2.14 | 1.12 | 1.14 | 1.87 | 2.07 | 2.14 |  |
| *sd* | *0.07* | *0.06* | *0.12* | *0.19* | *0.21* | *0.09* | *0.07* | *0.15* | *0.18* | *0.21* | *0.09* | *0.07* | *0.16* | *0.20* | *0.22* |  |
|  |  |  |  |  |  |  |  |  |  |  |  |  |  |  |  |  |

| **B- Fusion illusion** | | | | | | | | | | | | | | | | | | |
| --- | --- | --- | --- | --- | --- | --- | --- | --- | --- | --- | --- | --- | --- | --- | --- | --- | --- | --- |
|  |  | | | | | |  | | | | | |  | | | | | |
| **MwA** | **Baseline** | | | | | | **Cathodal** | | | | | | **Sham** | | | | | |
| Flashes/Beeps | **2/0** | **3/0** | **4/0** | **2/1** | **3/1** | **4/1** | **2/0** | **3/0** | **4/0** | **2/1** | **3/1** | **4/1** | **2/0** | **3/0** | **4/0** | **2/1** | **3/1** | **4/1** |
| Perceived flashes | 2.19 | 2.85 | 3.39 | 2.00 | 2.74 | 3.29 | 2.41 | 3.02 | 3.57 | 2.23 | 3.00 | 3.45 | 2.43 | 3.15 | 3.54 | 2.28 | 1.94 | 3.39 |
| *sd* | *0.14* | *0.14* | *0.13* | *0.12* | *0.14* | *0.18* | *0.15* | *0.14* | *0.12* | *0.13* | *0.14* | *0.18* | *0.15* | *0.14* | *0.12* | *0.13* | *0.16* | *0.18* |
|  |  |  |  |  |  |  |  |  |  |  |  |  |  |  |  |  |  |  |
| **MwoA** |  |  |  |  |  |  |  |  |  |  |  |  |  |  |  |  |  |  |
| Flashes/Beeps | **2/0** | **3/0** | **4/0** | **2/1** | **3/1** | **4/1** | **2/0** | **3/0** | **4/0** | **2/1** | **3/1** | **4/1** | **2/0** | **3/0** | **4/0** | **2/1** | **3/1** | **4/1** |
| Perceived flashes | 1.96 | 2.81 | 3.14 | 1.73 | 2.46 | 3.00 | 2.10 | 2.77 | 3.12 | 1.90 | 2.67 | 3.09 | 2.14 | 2.71 | 3.10 | 1.93 | 1.96 | 3.04 |
| *sd* | *0.14* | *0.14* | *0.13* | *0.12* | *0.14* | *0.18* | *0.15* | *0.14* | *0.12* | *0.13* | *0.14* | *0.18* | *0.15* | *0.14* | *0.12* | *0.13* | *0.16* | *0.18* |
|  |  |  |  |  |  |  |  |  |  |  |  |  |  |  |  |  |  |  |

**Table 1.** Mean values of perceived flashes for fission illusion condition (A) and fusion illusion condition (B) are shown. For each value stardard deviation of mean (sd) is reported. For other details see text in the method section.
